# Personalized Hearing Loss Care Using SNOMED CT-Aligned Ontology and Random Forest Machine Learning: A Hybrid Decision-Support Framework

**DOI:** 10.3390/audiolres16020037

**Published:** 2026-03-02

**Authors:** Darine Kebsi, Chamseddine Barki, Ismail Dergaa, Riadh Gouider, Halil İbrahim Ceylan, Amina Maddouri, Abderrazak Jemai, Mourad Elloumi, Nicola Luigi Bragazzi, Hanene Boussi Rahmouni

**Affiliations:** 1 Research Laboratory of Biophysics and Medical Technologies, The Higher Institute of Medical Technologies of Tunis, University of Tunis El Manar, 9, Street Z. Essafi, Tunis 1006, Tunisia; kebsidarine290@gmail.com (D.K.); chamseddine.barki@istmt.utm.tn (C.B.); hanene.boussi@istmt.utm.tn (H.B.R.); 2 High Institute of Sport and Physical Education of Ksar Said, University of Manouba, Mannouba 2010, Tunisia; phd.dergaa@gmail.com; 3 High Institute of Sport and Physical Education of El Kef, University of Jendouba, Jendouba 7100, Tunisia; 4 Neurology Department, LR18SP03, Clinical Investigation Center Neuroscience and Mental Health, Razi University Hospital, Tunis 2010, Tunisia; riadh.gouider@gnet.tn; 5 Faculty of Medicine of Tunis, University of Tunis El Manar, Tunis 1006, Tunisia; 6 Department of Physical Education of Sports Teaching, Faculty of Sports Sciences, Atatürk University, Erzurum 25240, Türkiye; 7 Department of R&D in Life Science and Chemicals, Expleo Groupe, 78180 Montigny le Bretonneux, France; amina.maddouri@gmail.com; 8 SERCOM-Lab., INSAT, Tunisia Polytechnic School, Carthage University, Tunis 1080, Tunisia; abderrazak.jemai@insat.rnu.tn; 9 Department of Computer Science, College of Computing and Information Technology, University of Bisha, Bisha 67714, Saudi Arabia; mourad.elloumi@gmail.com; 10 Laboratory for Industrial and Applied Mathematics (LIAM), Department of Mathematics and Statistics, York University, Toronto, ON M3J 1P3, Canada

**Keywords:** artificial intelligence, hearing loss, machine learning, medical informatics, ontology, personalized medicine, Random Forest, semantic reasoning, SNOMED CT

## Abstract

Background: Hearing loss affects over 466 million individuals globally and is recognized as a major risk factor for Alzheimer’s disease, yet treatment personalization remains limited due to the complexity and diversity of underlying causes. Current diagnostic and therapeutic approaches lack standardized methods to accurately predict the most appropriate intervention for individual patients. The integration of medical ontologies with machine learning offers a promising solution for enhancing diagnostic accuracy and treatment personalization. Aim: Our study aimed to (i) develop a Systematized Nomenclature of Medicine—Clinical Terms (SNOMED CT)-aligned clinical ontology for hearing loss using Semantic Web Rule Language for automated reasoning; (ii) implement a Random Forest classifier trained on ontology-enriched patient data to classify hearing loss types (conductive, sensorineural, mixed, or normal); and (iii) predict optimal personalized treatments based on laterality, severity, audiometric thresholds, and medical history using real-world patient data. Methods: We developed a task ontology using Protégé 5.6.3 with Web Ontology Language (OWL), integrated SNOMED CT terminology alignment, and implemented Semantic Web Rule Language rules executed by the Pellet 2.2.0 reasoner. The framework was trained and evaluated on 3723 adult patients from the 2015–2016 National Health and Nutrition Examination Survey (NHANES) dataset with complete audiometric and clinical data. Random Forest models were developed using an 80–20 train-test split with stratified sampling and five-fold cross-validation. Performance was compared between K-Means clustering-based labeling and ontology-based semantic inference using accuracy, precision, recall, F1-score, and log loss metrics. Results: The ontology successfully generated semantic labels for all 3723 patients, enabling precise classification of hearing loss types, severity levels, and laterality. The Random Forest model with K-Means clustering achieved a test accuracy of 90.2% with a log loss of 0.2766 and a cross-validation mean accuracy of 91.22% (standard deviation 1.2%). Integration of ontology-based semantic enrichment significantly improved performance, achieving a test accuracy of 92.48% with a cross-validation mean accuracy of 92.80% (standard deviation 0.9%). F1-scores improved across all classes, with mixed hearing loss showing a notable increase from 0.86 to 0.92. Feature importance analysis identified audiometric thresholds, ontology-derived severity labels, and medical history as top predictors, enhancing clinical interpretability. Conclusions: This study demonstrates that combining SNOMED CT-aligned ontology with Random Forest classification achieves superior diagnostic accuracy and enables personalized treatment recommendations for hearing loss. The hybrid framework provides clinically interpretable decision support while ensuring semantic interoperability with electronic health records. Multi-institutional validation studies are necessary to assess generalizability across diverse populations before clinical deployment.

## 1. Introduction

Hearing loss represents a significant global health challenge, affecting over 466 million individuals worldwide, with projections estimating an increase to 900 million by 2050 [1]. Multiple etiological factors contribute to hearing impairment, including aging-related degeneration [2,3], occupational and recreational noise exposure [4], ototoxic medication effects [5,6], chronic systemic illnesses such as diabetes mellitus and hypertension [7,8,9,10], genetic predispositions, and behavioral influences, including smoking and alcohol consumption [11,12,13]. This condition profoundly impacts multiple dimensions of daily life, including verbal communication abilities [14], social interaction patterns [15], educational and professional outcomes [16,17], and cognitive health, with established associations with dementia risk [18]. Effective and individualized interventions are essential to mitigate these multifaceted impacts [19]. However, existing therapeutic approaches, including conventional hearing aids and cochlear implants, frequently lack adequate personalization and accessibility, substantially limiting their clinical efficacy and patient satisfaction [20,21].

Beyond the sensory deficit itself, hearing loss affects quality of life across multiple domains. Verbal communication difficulties create barriers to social participation, while educational and professional success may be compromised by auditory processing challenges. Cognitive health is particularly concerning, as epidemiological evidence establishes hearing loss as one of the primary modifiable risk factors for Alzheimer’s disease and related dementias. Early diagnosis and timely intervention are therefore critical not only for auditory function but also for broader health outcomes. Despite these documented needs, many individuals do not receive adequate care due to multiple barriers: high costs of hearing devices, limited awareness of treatment options, insufficient access to specialized audiological services, and persistent stigma associated with hearing aid use. Moreover, existing interventions predominantly rely on generalized treatment protocols that inadequately account for patient-specific factors such as the unique combination of audiometric thresholds [22], medical comorbidities, and lifestyle characteristics [23]. This highlights a critical gap in current clinical practice: although personalized medicine has demonstrated potential to improve outcomes across medical disciplines [24], hearing loss management remains largely “one-size-fits-all” rather than individualized [25]. Addressing these limitations requires enhanced strategies that integrate semantic reasoning with predictive analytics to enable more precise, data-driven, and patient-centered audiology care.

Biomedical ontologies provide a structured framework for representing domain knowledge through formal concepts, hierarchical relationships, and logical axioms [26]. In biomedical informatics, ontologies are typically constructed using Web Ontology Language, ensuring logical formalization while maintaining compatibility with Semantic Web standards. A fundamental advantage of ontologies lies in their support for automated reasoning through inference engines that exploit axioms and relationships to derive new knowledge or verify logical consistency [27]. For example, a patient presenting specific symptom combinations can be automatically classified into diagnostic categories even when not explicitly specified in the knowledge base. Ontologies have been successfully applied across diverse medical domains, including bladder cancer treatment, where OWL-based ontologies can structure complex patient and therapy knowledge to predict side effects and support clinical decisions [28]. Similarly, in radiation oncology, ontologies model treatment pathways and evidence-based guidelines, enabling rule-based reasoning to anticipate complications and adjust therapy plans [29]. Reference medical ontologies such as SNOMED CT [30] and the Foundational Model of Anatomy [31] facilitate semantic interoperability and evidence-based decision-making across heterogeneous healthcare information systems. These applications demonstrate that ontologies ensure data consistency, promote terminological standardization, enhance semantic interoperability [32], and support computer-assisted clinical decision-making by providing structured knowledge that intelligent algorithms can process to improve patient care.

Artificial intelligence is playing an increasingly important role in medicine due to its capacity to analyze large volumes of complex data and assist healthcare professionals in clinical decision-making [33]. Machine learning, a core branch of artificial intelligence, encompasses algorithms that enable computer systems to automatically learn patterns from data without explicit programming for each task [34]. These techniques have progressively transformed numerous medical fields, including audiology. Early approaches based on rigid expert systems with predefined rules, while useful, were limited by their inability to handle novel cases or adapt to emerging trends in auditory data. Contemporary machine learning and deep learning methods enable analysis of large medical datasets and extraction of clinically relevant information for diagnosing and managing hearing disorders [35]. These technologies provide valuable clinical decision support by automatically classifying patients based on hearing loss type (sensorineural, conductive, mixed, or normal hearing), recommending tailored personalized treatments (hearing aids, cochlear implants, or other interventions), and analyzing audiograms and tympanograms using models capable of detecting complex patterns imperceptible to human observation.

Despite significant advances in artificial intelligence and machine learning-based applications in healthcare, these approaches possess inherent limitations, particularly regarding interpretability and the capacity to integrate explicit clinical knowledge. Machine learning models, while effective at detecting complex patterns in data, frequently function as opaque systems (“black boxes”) where decision-making processes remain difficult to understand or explain. To address these limitations, the integration of ontologies into artificial intelligence systems enables the development of hybrid models that combine the predictive power of machine learning with the semantic richness and logical structure of formalized medical knowledge [36]. This integration contextualizes data, improves result consistency, and provides more comprehensible explanations for healthcare professionals. The primary advantages of hybrid approaches include enhanced interpretability of results through explanation of decisions based on explicit medical knowledge, contextualization of predictions by accounting for complex relationships between medical concepts, increased robustness when handling uncertainty and incomplete data through ontology-based logical reasoning, and improved feature engineering by facilitating identification of clinically significant characteristics.

Although several studies have applied artificial intelligence to audiology, significant research gaps persist. Tomiazzi et al. demonstrated the efficacy of machine learning algorithms in classifying hearing impairment among Brazilian farmers exposed to pesticides and cigarette smoke [6], but their approach relied solely on statistical methods without a semantic structure. AlSamhori et al. explored artificial intelligence for hearing loss prevention and management [37], emphasizing predictive models, but lacked integration with standardized medical taxonomies. The Hearing Impairment Ontology developed by Hotchkiss et al. provides a foundational framework for unifying hearing loss knowledge [38] but primarily focuses on knowledge representation rather than predictive analytics and lacks alignment with clinical terminologies like SNOMED CT, which can hinder interoperability. Recent hybrid approaches have shown promise in other medical domains: El Massari et al. utilized ontology-based machine learning to predict diabetes outcomes [39], while Piriou et al. developed knowledge graphs for leukemia data representation [40]. However, to our knowledge, no study has combined SNOMED CT-aligned clinical ontology with machine learning specifically for personalized hearing loss diagnosis and treatment prediction on a large-scale patient dataset.

Based on these identified research gaps, our study aimed to (i) develop and validate a comprehensive clinical ontology for hearing loss aligned with SNOMED CT terminology using Semantic Web Rule Language rules for automated reasoning; (ii) implement a hybrid framework integrating ontology-based semantic enrichment with Random Forest machine learning classification for automated diagnosis of hearing loss types (conductive, sensorineural, mixed, or normal); and (iii) provide personalized treatment recommendations based on audiometric thresholds, laterality patterns, severity classifications, and patient medical history using real-world data from 3723 adult patients.

## 2. Materials and Methods

### 2.1. Ethical Approval

This study utilized de-identified publicly available data from the National Health and Nutrition Examination Survey 2015–2016 cycle, which received approval from the National Center for Health Statistics Research Ethics Review Board. All data were collected in accordance with ethical standards for research with human subjects. As this study involved analysis of existing de-identified data without direct patient contact, additional institutional review board approval was not required.

### 2.2. Data Acquisition and Preprocessing

We utilized the 2015–2016 National Health and Nutrition Examination Survey dataset, selected for its comprehensive audiometric and clinical data from adult patients. This version was chosen because it contains the most complete set of audiological variables and the lowest rate of missing values among the available National Health and Nutrition Examination Survey datasets. The original data files contained different participant counts across various modules, necessitating a preprocessing phase to merge multiple sources and harmonize records using patient identifiers.

During the initial data exploration phase, common preprocessing strategies for missing values, such as mean imputation for continuous variables (hearing thresholds, age) and mode imputation for categorical variables (medical conditions, ear examination findings), were considered. However, these approaches were not implemented for the analyses reported in this study. Given the sensitivity of audiometric thresholds to distributional distortions and the potential propagation of bias in both ontology-based inference and machine learning models, a complete-case analysis was adopted. Consequently, patients presenting missing audiometric values were excluded prior to ontology reasoning and machine learning experiments, and all reported results are based exclusively on records with complete data. This choice was motivated by the need to preserve the integrity of audiometric patterns and avoid artificial smoothing effects that may arise from simplistic imputation strategies. The impact of advanced imputation techniques (for example, multiple imputation or model-based approaches) and associated sensitivity analyses is considered a relevant direction for future work.

The resulting dataset contained 3723 patients, integrating audiometric measurements (pure-tone thresholds at 500 Hz, 1000 Hz, 2000 Hz, 3000 Hz, 4000 Hz, 6000 Hz, and 8000 Hz for both ears), demographic variables (age, sex/gender, ethnicity), medical history (diabetes, hypertension, cardiovascular disease, noise exposure), and lifestyle factors (smoking status, alcohol consumption) without missing values. The dataset was suitable for personalized classification and modeling, but was initially unlabeled, requiring the implementation of a labeling strategy.

### 2.3. Data Labeling Strategies

To create a labeled dataset from the initially unlabeled National Health and Nutrition Examination Survey data, we employed two complementary strategies: unsupervised K-Means clustering for statistical segmentation and ontology-based inference for semantic labeling. K-Means clustering is used solely as an unsupervised baseline to segment patients in the initially unlabeled dataset. This comparison allows us to evaluate the added value of ontology-based semantic labeling. K-Means grouped patients according to hearing characteristics based on audiometric thresholds and tympanometric results.

The SNOMED CT-aligned ontology assigned precise clinical labels for hearing loss type (conductive, sensorineural, mixed, or normal), severity classification (normal, mild, moderate, severe, profound), laterality patterns (bilateral, unilateral left, unilateral right), and treatment recommendations (hearing aids, cochlear implants, auditory rehabilitation, surgical intervention). Importantly, ontology inference is performed exclusively on raw clinical and audiometric inputs, and no variables encoding hearing loss type, severity, laterality, or treatment are used during ontology reasoning. The ontology-derived concepts, which represent intermediate clinical features, are included alongside raw demographic, medical, and audiometric parameters as input features for the Random Forest model. The final target labels (hearing loss type, severity, laterality, and treatment) are used exclusively as outputs for supervised learning. This hybrid approach leverages ontology-based reasoning to enrich the feature set while avoiding circular dependency between feature representation and label generation.

These complementary methods enrich the dataset with meaningful clinical features, enhancing its utility for clinical decision-making and machine learning model training. The comparison of these two approaches aimed to demonstrate the added value of combining knowledge structuring through ontology with machine learning techniques for identifying coherent and clinically interpretable patient profiles.

### 2.4. K-Means Clustering for Unsupervised Patient Segmentation

As previously mentioned, we applied the K-Means algorithm, an unsupervised learning method that partitions data into clusters based on feature similarity [41]. The objective was to classify patients according to three essential clinical criteria: type of hearing loss, severity level, and laterality pattern, subsequently enabling appropriate treatment recommendations for each case. The algorithm was applied directly to the dataset containing audiometric thresholds, tympanogram quality measurements, medical conditions, and other relevant clinical features. To determine the optimal number of clusters (k) for each classification task, we utilized the Elbow method and silhouette coefficient analysis [42]. These complementary techniques evaluate clustering quality and identify the k-value that optimally separates patient profiles. For hearing loss type classification, k was set to 4 (conductive, sensorineural, mixed, and normal hearing), based on both the Elbow method results and established domain knowledge from audiology clinical practice. For severity classification, k was set to 5 categories (normal, mild, moderate, severe, profound) following standard audiometric classification schemes. Laterality classification employed k equals 4 (normal hearing, bilateral hearing loss, unilateral left hearing loss, unilateral right hearing loss). The K-Means algorithm was then applied independently to feature sets corresponding to each of the three classification tasks. Following cluster assignment, results were merged into a consolidated dataset integrating all classification dimensions to provide a comprehensive view of each patient’s hearing profile. This integrated dataset facilitated subsequent clinical decision-making and enabled the development of personalized treatment recommendations.

### 2.5. Ontology Development and Architecture

Using an ontology for patient classification improves data quality and reliability by formally structuring concepts and their relationships within a specific domain [43]. Unlike purely statistical clustering approaches, ontology relies on a logical framework that facilitates inference and automatic categorization of patients based on their audiometric and clinical characteristics through rule-based reasoning. In this project, we developed a task ontology using Protégé 5.6.3, an open-source ontology editor supporting Web Ontology Language 2. While domain ontologies focus on representing general knowledge about a field, task ontologies are specifically designed to support particular activities or problem-solving processes within a domain. Our task ontology focuses on classifying patients according to hearing loss characteristics and recommending appropriate clinical interventions. The ontology architecture includes several hierarchically organized key classes: Anatomy (encompassing anatomical components of the auditory system including external ear, middle ear, inner ear, and auditory neural pathways), Hearing_Condition (with subclasses Hearing_Normal and Hearing_Loss, the latter further subdivided into Conductive_HL, Sensorineural_HL, and Mixed_HL), Types (capturing etiological categories), Laterality (distinguishing Bilateral, Unilateral_Left, and Unilateral_Right patterns), Severity (ranging from Normal to Mild, Moderate, Severe and Profound), Diagnostics (including audiometric tests, tympanometry, and otoacoustic emissions), and Treatments (encompassing hearing aids, cochlear implants, bone-anchored hearing systems, auditory rehabilitation, and surgical interventions). The semantic relationships between these classes (Has_Type, Has_Laterality, Has_Severity, Requires_Treatment) formally define how patient data are conceptually interconnected within the knowledge base.

Patient data corresponding to the 3723 individuals in the dataset, along with their medical and audiometric information, were introduced into the ontology automatically using the Owlready2 library in Python 3.10 [44]. This automated process enabled importing an unlabeled dataset while dynamically creating individuals (instances) corresponding to patients and populating their attributes with clinical data. Table 1 represents the ontology overview, demonstrating its comprehensive structure.

The ontology is organized around domain-level classes representing general hearing-related concepts (Anatomy, Hearing_Condition, Diagnostics, Therapies) and task-level elements supporting classification and clinical decision-making. It includes 27 object properties describing semantic relationships between entities (has_Type, has_Severity, has_Treatment, affects_Ear) and 39 data properties linking individuals to numerical or categorical attributes (hearing thresholds at specific frequencies, age, presence of medical conditions such as diabetes or hypertension). The substantial number of 145,683 axioms reflects the logical definitions, class restrictions, and inference rules that ensure data consistency and enable automated reasoning. The 3723 individuals correspond to patients imported from the National Health and Nutrition Examination Survey dataset, with each instance enriched with demographic, audiometric, tympanometric, and medical history information. Together, these components form a comprehensive knowledge base supporting both semantic data integration and intelligent inference for personalized hearing loss care.

**Figure 1:**
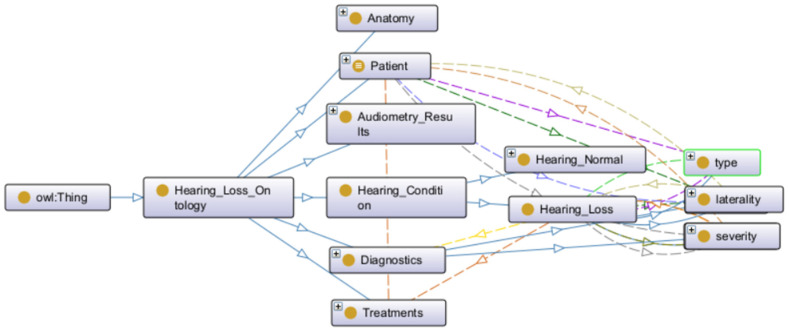
Overview of the hearing loss ontology structure.

Figure 1 represents an overview of the hearing loss ontology structure, illustrating the hierarchical organization of classes, subclasses, and selected relationships that support semantic reasoning and patient classification. Due to the ontology’s complexity with 145,683 axioms and extensive class hierarchies, a complete visualization of all components is not feasible within the manuscript constraints. The figure depicts the primary conceptual organization, including the Hearing_Condition class hierarchy, anatomical classifications, severity gradations, laterality patterns, and treatment categories, demonstrating how these elements interconnect to support clinical decision-making.

**Figure 2:**
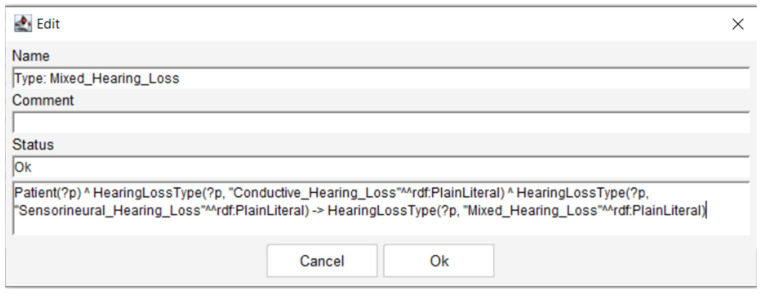
Mixed Hearing Loss rule.

Once the dataset was integrated into the ontology framework, we transformed a simple relational database into a comprehensive knowledge base comprising a fact base (containing patient data represented as individuals with assigned properties) and a rule base constructed with Semantic Web Rule Language. Semantic Web Rule Language rules were implemented to automate reasoning processes and classify patients according to their clinical characteristics [45]. For example, a Semantic Web Rule Language rule was implemented to infer cases of mixed hearing loss when both conductive and sensorineural characteristics are detected in the same patient, as represented in Figure 2. This rule specifies that any patient exhibiting both conductive and sensorineural hearing loss characteristics should be automatically assigned to the Mixed_Hearing_Loss class. Similar rules were developed for severity classification (assigning Profound_HL when pure-tone average exceeds 90 dB HL), laterality determination (classifying as Bilateral when both ears demonstrate hearing loss), and treatment recommendations (suggesting cochlear implant candidacy when severe-to-profound sensorineural hearing loss is present bilaterally with limited hearing aid benefit).

Figure 2 represents the Semantic Web Rule Language rule for mixed hearing loss inference, demonstrating how logical axioms enable automatic classification based on clinical patterns.

We utilized the Pellet 2.2.0 reasoner, a powerful Description Logic inference engine compatible with Web Ontology Language 2 ontologies [46], to execute reasoning and infer new knowledge from explicit facts and logical rules. The Pellet reasoner performs classification (computing class hierarchies), consistency checking (verifying logical coherence), and realization (determining class membership for individuals). Finally, the Owlready2 Python library was employed to extract the inferred results from the reasoned ontology and generate a final labeled dataset in CSV format, suitable for machine learning model training.

### 2.6. SNOMED CT Alignment for Semantic Interoperability

**Figure 3:**
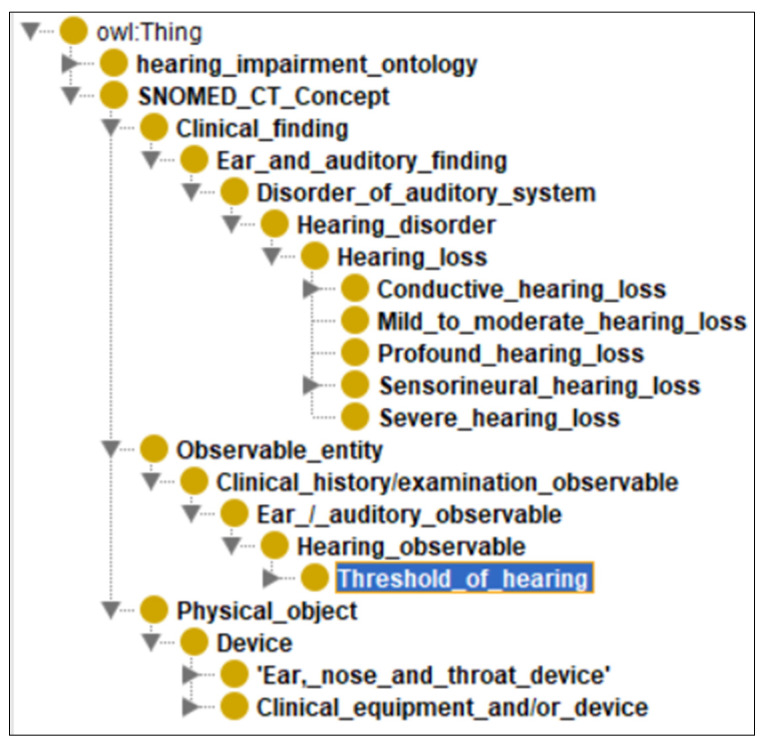
Embedding SNOMED CT structure in the hearing loss ontology.

To enhance semantic interoperability and ensure consistency with established clinical terminologies, key concepts in the hearing loss ontology were aligned with SNOMED CT. SNOMED CT (Systematized Nomenclature of Medicine Clinical Terms) represents the most comprehensive multilingual clinical healthcare terminology worldwide and contains over 350,000 active concepts covering clinical findings, procedures, body structures, organisms, substances, and pharmaceuticals. The hierarchical structure of SNOMED CT was incorporated directly into the SNOMED_CT_Concept class within our ontology, effectively embedding its conceptual framework into the hearing loss knowledge base, as illustrated in Figure 3. This alignment enables the ontology to harmonize patient data with widely recognized medical standards and supports integration with diverse audiology information systems and electronic health records.

Figure 3 represents the embedding of the SNOMED CT structure in the hearing loss ontology, demonstrating how standardized medical terminology is integrated to ensure interoperability.

During the mapping process, concepts from our hearing loss ontology were matched to corresponding SNOMED CT codes when available. For example, “Sensorineural hearing loss” was mapped to SNOMED CT concept 60700002, “Conductive hearing loss” to 44057008, “Mixed hearing loss” to 36885005, and specific severity levels and laterality patterns to their respective codes. No direct logical conflicts arose between the hearing loss ontology and SNOMED CT, since the SNOMED CT hierarchy served as a reference framework rather than imposing strict one-to-one correspondences that might conflict with our domain-specific classifications. Concepts for which no matching SNOMED CT code exists (such as certain treatment recommendation categories or specific audiometric threshold combinations) were retained in the ontology to preserve completeness, allowing for future refinement as the SNOMED CT terminology evolves and expands its coverage of audiology-specific concepts.

### 2.7. Random Forest Classification Architecture

We employed Random Forest, an ensemble learning method where multiple decision trees are trained independently on bootstrapped samples of the data and their predictions are aggregated to improve model robustness and reduce overfitting [47]. Random Forest is a supervised machine learning technique particularly well-suited for heterogeneous medical data. Implementation was performed using Python 3.10 with the scikit-learn library on Google Colaboratory computing infrastructure. In this project, the Random Forest classifier was designed to predict hearing loss types and treatment recommendations based on variables generated by both labeling approaches: K-Means clustering and ontological inference. The model processes both raw clinical characteristics (age, medical history including diabetes and hypertension, audiometric thresholds at multiple frequencies) and ontology-enriched variables (Has_Type, Has_Laterality, Has_Severity, recommendedTreatment). Random Forest was selected for its ability to efficiently handle heterogeneous data types, including both numerical features (audiometric thresholds measured in decibels) and categorical variables (medical history, laterality patterns), its inherent robustness against overfitting through ensemble averaging, and its transparency regarding feature importance, which is essential in clinical settings for interpretability and trust [48].

The dataset was partitioned into training (80%) and testing (20%) subsets using stratified sampling to ensure balanced representation of all hearing loss classes in both sets. The Random Forest model was trained on the labeled training set to predict hearing loss types (normal, conductive, sensorineural, mixed) and appropriate treatment recommendations based on input features, including age, bilateral audiometric thresholds, tympanometric results, medical comorbidities, and ontology-derived semantic labels when applicable.

### 2.8. Model Evaluation and Hyperparameter Optimization

Random Forest model evaluation relied on multiple complementary techniques to analyze performance and optimize hyperparameters for improved generalization. First, confusion matrices were constructed to evaluate the model’s ability to correctly classify different hearing loss categories, enabling calculation of class-specific precision (positive predictive value), recall (sensitivity), and F1-score (harmonic mean of precision and recall) [49]. These metrics are essential to verify how accurately the model predicts hearing loss types, severity levels, and laterality patterns, which are critical for proposing personalized treatments. Second, hyperparameter optimization was performed to improve model performance and reduce overfitting risk. Key parameters including n_estimators (number of trees in the ensemble, tested range: 100–500), max_depth (maximum depth of individual trees, tested range: 10–50), min_samples_split (minimum samples required to split an internal node, tested range: 2–20), and max_features (number of features considered for each split, tested values: ‘sqrt’, ‘log2’, and specific proportions) were systematically adjusted through grid search to ensure optimal balance between model complexity and predictive performance. Third, five-fold cross-validation was applied to ensure model robustness and avoid overfitting [50]. The dataset was divided into five distinct subsets, with the model trained on four folds and tested on the remaining fold at each iteration, repeating this process five times. This approach provides a more reliable assessment of model performance across different data partitions, ensuring generalization capability to unseen data. Model performance was evaluated using accuracy, precision, recall, F1-score, and log loss, with results reported separately for K-Means-based and ontology-based labeling approaches.

### 2.9. Statistical Analysis

Performance comparison between K-Means clustering and ontology-based labeling was conducted using descriptive statistics for classification metrics. Mean accuracy and standard deviation from five-fold cross-validation provided estimates of model stability and generalization performance. Log loss was calculated to assess prediction probability calibration. Feature importance was extracted from the trained Random Forest models to identify the most influential predictors for hearing loss classification and treatment recommendation. All statistical analyses and visualizations were performed using Python scientific computing libraries, including NumPy, pandas, scikit-learn, and matplotlib.

## 3. Results

### 3.1. Ontology-Based Inference Results

The hearing loss ontology, structured according to SNOMED CT terminology alignment and enhanced with Semantic Web Rule Language rules, was executed using the Pellet 2.2.0 reasoner and successfully generated semantic labels for all 3723 patients in the dataset. The ontology enabled precise automated classification of hearing loss types (normal, conductive, sensorineural, mixed), severity levels (normal, mild, moderate, severe, profound), laterality patterns (bilateral, unilateral left, unilateral right), and treatment recommendations (hearing aids, cochlear implants, auditory rehabilitation, surgical intervention) based on both audiometric thresholds and clinical history. The integration of ontological reasoning allowed the system to infer additional properties that were not explicitly present in the raw dataset, thereby enriching the data and substantially enhancing its clinical utility for downstream machine learning tasks.

**Figure 4:**
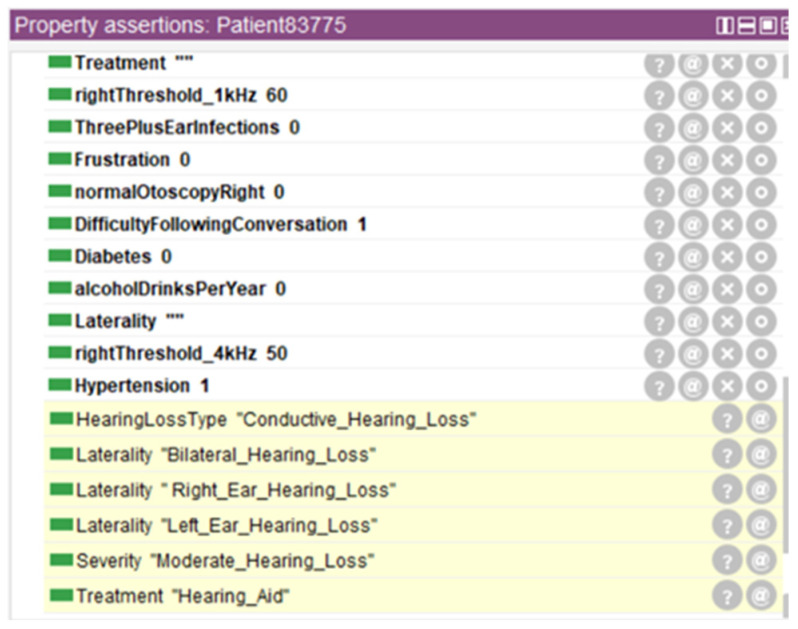
Inferred properties for Patient83775.

Figure 4 represents inferred properties for a randomly selected patient (Patient83775), illustrating treatment suitability classifications and other clinically relevant attributes derived through ontological reasoning. The figure demonstrates how the reasoning engine automatically assigned multiple inferred properties, including hearing loss type classification, severity grading, laterality determination, and treatment recommendation, based on the patient’s audiometric profile and medical history. These inferred properties were not present in the original dataset but were automatically generated through application of Semantic Web Rule Language rules and Description Logic reasoning.

**Figure 5:**
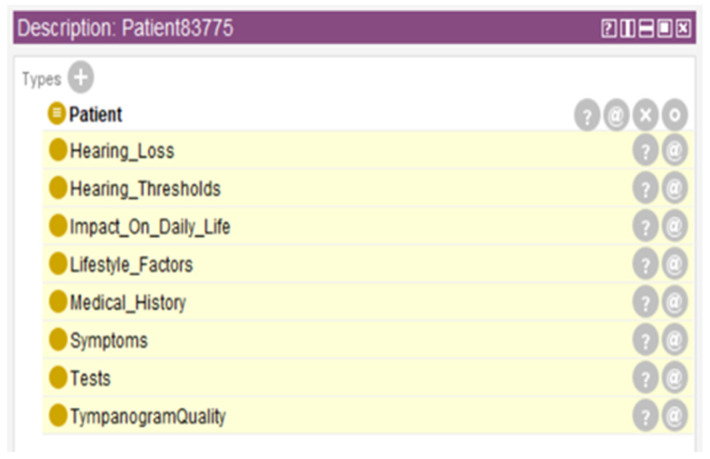
Inferred class hierarchy for Patient83775.

Figure 5 represents the ontology’s inferred class hierarchy for Patient83775, with automatically classified classes highlighted. This visualization demonstrates how the reasoning engine places individual patients into appropriate classification categories based on their clinical characteristics. Although these figures focus on a single illustrative patient example, they demonstrate the ontology’s capability to automatically enrich patient records systematically, generate structured and clinically interpretable semantic labels, and support downstream predictive modeling. This ontological reasoning approach operates consistently across the entire dataset, providing both detailed individual patient insights and a scalable framework for large-scale patient classification without requiring manual visualization of results for every individual.

### 3.2. Random Forest Classification with K-Means Clustering

When paired with K-Means clustering for initial data labeling, the Random Forest model achieved an overall test set accuracy of 90.2%, reflecting the proportion of correctly classified instances across all hearing loss categories, with a log loss of 0.2766. Table 2 represents the detailed performance metrics by hearing loss class. Precision ranged from 0.85 to 0.91 across classes, with sensorineural hearing loss demonstrating the highest precision (0.91), reflecting accurate positive predictions. Recall values ranged from 0.86 to 0.90, with conductive hearing loss achieving the highest recall (0.90), indicating effective identification of true positive cases. F1-scores, the harmonic mean of precision and recall, ranged from 0.85 to 0.91, with sensorineural hearing loss showing the highest F1-score (0.91) due to its higher prevalence in the dataset, which provided more training examples for this class. Normal hearing and mixed hearing loss classes demonstrated slightly lower F1-scores (0.85 and 0.86, respectively), reflecting the challenges in correctly classifying these categories using statistical clustering alone.

Five-fold cross-validation yielded a mean accuracy of 91.22% with a standard deviation of 1.2%, confirming stable and consistent performance across different data partitions. The slight difference between cross-validation mean accuracy (91.22%) and test set accuracy (90.2%) likely reflects natural variability in the specific composition of the held-out test set or the presence of more challenging classification cases in that particular partition. Although overall accuracy was high, the log loss of 0.2766 indicates that predicted probabilities were sometimes not perfectly calibrated, meaning the model occasionally exhibited high confidence in predictions that were not always correct or, conversely, lower confidence for some correct predictions.

### 3.3. Random Forest Classification with Ontology-Based Enrichment

Integrating ontology-based semantic enrichment significantly enhanced Random Forest model performance, achieving a test set accuracy of 92.48% with a log loss of 0.3842. Table 3 represents detailed performance metrics demonstrating improvements across all hearing loss classes. Precision values improved to a range of 0.89 to 0.94 across classes, with normal hearing achieving the highest precision (0.94). Recall increased to a range of 0.90 to 0.93, with sensorineural hearing loss demonstrating the highest recall (0.93). F1-scores improved substantially across all classes, ranging from 0.89 to 0.94. Notably, mixed hearing loss showed a substantial F1-score increase from 0.86 (K-Means approach) to 0.92 (ontology approach), demonstrating the ontology’s particular value in correctly identifying complex hearing loss patterns that involve both conductive and sensorineural components.

The higher log loss (0.3842) despite improved accuracy is not contradictory but rather reflects the fact that while the model made more correct predictions overall, the predicted class probability distributions were less sharply confident. This pattern likely results from the introduction of semantic features that increased model complexity and introduced greater uncertainty for some borderline instances where clinical characteristics were ambiguous. Cross-validation yielded a mean accuracy of 92.80% with a standard deviation of 0.9%, demonstrating both improved performance and enhanced stability compared to the K-Means approach. The lower standard deviation indicates more consistent performance across different data folds, suggesting better generalization capability.

### 3.4. Feature Importance Analysis

Feature importance analysis from the ontology-enriched Random Forest model revealed that audiometric thresholds at specific frequencies (particularly 4000 Hz and 6000 Hz bilaterally) were the most influential predictors, consistent with audiological knowledge that high-frequency hearing loss is common in sensorineural patterns. Ontology-derived features, including severity classification labels and laterality patterns, ranked among the top ten most important features, demonstrating the value of semantic enrichment. Medical history variables, including diabetes diagnosis, hypertension, and noise exposure history, also contributed substantially to classification accuracy. Age was a moderately important feature, reflecting the progressive nature of age-related hearing loss. The feature importance distribution was relatively balanced rather than dominated by a small number of features, suggesting that the model successfully integrated multiple complementary sources of information.

### 3.5. Comparative Performance Summary

Table 4 represents the comprehensive comparison between Random Forest with K-Means clustering and Random Forest with ontology-based enrichment. The ontology-based approach demonstrated superior performance across all evaluation metrics. Test accuracy improved by 2.28 percentage points (from 90.2% to 92.48%), representing a relative improvement of approximately 2.5%. Cross-validation mean accuracy increased from 91.22% to 92.80%, with standard deviation decreasing from 1.2% to 0.9%, indicating both improved performance and enhanced stability. F1-scores improved for all hearing loss classes, with the most substantial gains observed for mixed hearing loss (6 percentage point improvement) and normal hearing (4 percentage point improvement), classes that presented the greatest challenges for purely statistical clustering approaches. While log loss increased from 0.2766 to 0.3842, this reflects more conservative probability estimates rather than degraded performance, as the actual classification decisions improved substantially.

The differences between test set accuracy and cross-validation accuracy for both approaches highlight the natural variability inherent in specific test set compositions rather than indicating overfitting. Both models demonstrated consistent performance across cross-validation folds, with the ontology-based approach showing superior stability (lower standard deviation).

Overall, semantic enrichment through ontology-based reasoning provided more informative and clinically meaningful features, enabling accurate differentiation of rare or complex hearing loss cases that were challenging for unsupervised clustering methods alone.

## 4. Discussion

This study presents a hybrid framework combining SNOMED CT-aligned clinical ontology with Random Forest machine learning to enhance hearing loss diagnosis and treatment personalization. By analyzing data from 3723 National Health and Nutrition Examination Survey adult patients, the ontology-enriched Random Forest model achieved a classification accuracy of 92.48%, providing reliable identification of hearing loss types (conductive, sensorineural, mixed, or normal) and enabling personalized intervention recommendations based on audiometric profiles, laterality patterns, severity classifications, and medical history. The following sections reflect on the significance of these results, compare performance to existing approaches in the literature, discuss clinical implications, and acknowledge limitations while identifying future research directions.

### 4.1. Comparison Between Random Forest with K-Means and Random Forest with Ontology

Integrating ontology-based semantic enrichment into the Random Forest model substantially improved classification performance compared to K-Means clustering-based labeling. While Random Forest combined with K-Means produced respectable results with 90.2% accuracy, its reliance on purely statistical clustering patterns sometimes overlooked nuanced clinical relationships and pathophysiological mechanisms, particularly for complex cases such as mixed hearing loss. Statistical clustering groups patients based on numerical similarity in feature space without incorporating domain knowledge about the clinical significance of specific audiometric patterns or the etiological relationships between different types of hearing impairment. In contrast, Random Forest with ontology-based enrichment incorporated a semantically richer knowledge structure that explicitly captures complex relationships between clinical variables, audiometric measurements, and underlying pathophysiology. This semantic enrichment resulted in higher test accuracy (92.48% versus 90.2%), improved F1-scores across all classes (particularly for mixed hearing loss, which improved from 0.86 to 0.92), and enhanced cross-validation stability (standard deviation 0.9% versus 1.2%). The ontology provided structured domain knowledge that guided feature engineering and enabled the model to recognize clinically meaningful patterns that pure statistical methods might miss. Adding a formalized knowledge base and ontology to a machine learning model enriches training data by providing semantic structure that organizes and connects information coherently. This facilitates integration of expert clinical knowledge, improves model interpretability by clarifying relationships between concepts, and enables better handling of heterogeneous and incomplete data. Additionally, ontology helps formalize clinical rules and diagnostic axioms, leading to more accurate predictions tailored to domain-specific requirements. It also provides superior management of uncertainty and ambiguity, thereby increasing the robustness and reliability of generated results.

### 4.2. Comparative Analysis with Existing Classification Approaches

Our results compare favorably to existing studies in audiology and medical informatics. Tomiazzi et al. applied machine learning to classify hearing impairment in Brazilian farmers exposed to pesticides, achieving approximately 85% accuracy [6], but their approach lacked semantic structure and domain knowledge integration, limiting interpretability and potentially missing complex clinical patterns. Similarly, Kassjański et al. developed an automated hearing loss type classification system based on pure-tone audiometry data, reporting accuracies between 85% and 91% depending on the classification algorithm employed [51], comparable to our K-Means baseline but inferior to our ontology-enriched approach. Cárdenas et al. reported over 90% accuracy using synthetic brainstem auditory evoked potential data and artificial neural networks [52]. Although their accuracy is comparable to our results, their model was developed using synthetic rather than real patient data, potentially limiting clinical applicability and generalizability to diverse patient populations encountered in actual clinical practice.

Recent deep learning approaches have also been explored for hearing loss applications. Seo et al. employed a multilayer perceptron combined with a meta-classifier to predict hearing recovery in 1108 patients with idiopathic sudden sensorineural hearing loss, achieving an area under the receiver operating characteristic curve of 0.922 [53]. While demonstrating the potential of purely data-driven deep learning models for specific prognostic tasks, our hybrid ontology-Random Forest model attains higher classification accuracy (92.48%) for the broader task of multi-class hearing loss type classification and, critically, integrates semantic reasoning with domain knowledge to provide clinical interpretability. This interpretability is essential for clinical adoption, as healthcare professionals require an understanding of why a particular classification or treatment recommendation was generated. Compared to ontology-only systems such as the Hearing Impairment Ontology [38], which focus primarily on knowledge representation and terminology standardization without predictive capabilities, our approach adds actionable predictive power while maintaining semantic transparency and clinical interpretability. These comparisons highlight our hybrid model’s unique combination of high classification accuracy, semantic interpretability through ontology alignment, and clinical actionability, effectively bridging the gap between structured domain knowledge and data-driven predictive analytics for practical clinical decision support.

### 4.3. Clinical Implications and Practical Applications

The hybrid ontology-Random Forest framework provides clinicians with actionable and interpretable insights for hearing loss management. Feature importance analysis identified audiometric thresholds (particularly at 4000 Hz and 6000 Hz frequencies where sensorineural damage typically manifests earliest) and ontology-derived semantic labels (severity classifications and laterality patterns) as the most influential predictors, supporting evidence-based clinical decision-making grounded in established audiological knowledge. Medical history variables, including diabetes mellitus, hypertension, and occupational noise exposure, also contributed substantially to classification accuracy, reflecting the multifactorial etiology of hearing loss documented in the epidemiological literature [7,8,9]. By integrating semantic reasoning with machine learning, the model enables the development of tailored treatment plans that reflect each patient’s unique clinical profile, moving beyond one-size-fits-all approaches toward true personalized medicine in audiology care.

The alignment with SNOMED CT terminology provides semantic interoperability with electronic health record systems [30], facilitating seamless adoption across diverse clinical settings and enabling automatic data exchange between audiology information systems, primary care records, and specialty consultations. This interoperability is particularly valuable in integrated healthcare delivery systems where hearing loss management involves coordination between audiologists, otolaryngologists, primary care physicians, and potentially geriatricians or neurologists when cognitive concerns are present. In comparison, approaches based solely on K-Means clustering or other unsupervised methods, while useful for exploratory data analysis and initial patient segmentation, tend to oversimplify complex clinical cases by relying purely on statistical similarity without incorporating pathophysiological understanding. The ontology’s critical role in capturing nuanced clinical patterns, such as distinguishing between different etiologies of sensorineural hearing loss or recognizing subtle mixed conductive-sensorineural components, substantially enhances diagnostic accuracy and clinical utility.

The model could be deployed in clinical audiology practice as a decision-support tool that processes patient audiometric data, medical history, and demographic information to provide real-time classification of hearing loss type and evidence-based treatment recommendations. This could reduce diagnostic variability, support less experienced clinicians in complex cases, facilitate standardized documentation, and potentially improve access to audiological care in underserved areas where specialist expertise is limited. The transparent feature importance rankings enable clinicians to understand which factors most influenced a particular recommendation, building trust and facilitating informed shared decision-making with patients.

### 4.4. Technical Considerations and Model Performance

The higher log loss observed in the ontology-based model (0.3842 versus 0.2766 for K-Means) warrants discussion, as it might initially appear contradictory to the improved classification accuracy. Log loss penalizes confident but incorrect predictions more severely than uncertain predictions and rewards well-calibrated probability [54]. The increased log loss despite superior accuracy indicates that the ontology-enriched model, while making more correct final classifications, assigned more conservative and distributed probability estimates across classes rather than highly confident predictions. This pattern reflects increased model complexity introduced by semantic features, which captures more nuanced clinical information but also introduces greater uncertainty in borderline cases where clinical characteristics are ambiguous or overlapping. From a clinical perspective, this conservative probability estimation may actually be desirable, as it reflects appropriate epistemic humility in uncertain cases and could prompt clinicians to conduct additional diagnostic testing or consider multiple diagnostic possibilities rather than over-relying on a single confident but potentially incorrect automated classification.

The consistency between test set performance (92.48% accuracy) and cross-validation performance (92.80% mean accuracy with 0.9% standard deviation) provides strong evidence of model generalization capability without overfitting. The narrow standard deviation in cross-validation indicates stable performance across different data partitions, suggesting that the model has learned generalizable patterns rather than memorizing training set idiosyncrasies. The slight difference between the test accuracy and the cross-validation mean likely reflects natural sampling variability in the specific test set composition rather than systematic performance degradation on unseen data.

### 4.5. Ontology Design and Semantic Reasoning Advantages

The task ontology design, focusing on classification and treatment recommendation rather than comprehensive domain knowledge representation, provided an optimal balance between expressiveness and computational tractability. Task ontologies differ from domain ontologies by emphasizing problem-solving processes rather than exhaustive knowledge coverage [43]. This focused approach enabled efficient reasoning over 3723 patient instances without excessive computational burden while maintaining sufficient semantic richness to capture clinically relevant relationships. The Semantic Web Rule Language rules implemented in the ontology automated complex diagnostic reasoning that would traditionally require expert clinical judgment. For example, the mixed hearing loss inference rule automatically identified patients presenting both air-bone gaps (indicating conductive component) and reduced bone conduction thresholds (indicating sensorineural component), a pattern that requires careful interpretation of audiometric data and may be overlooked in rapid clinical assessments or by purely statistical classification methods.

Pellet 2.2.0 reasoner’s Description Logic inference capabilities enabled not only explicit rule application but also implicit classification through logical subsumption and property inheritance [46]. This allowed the system to infer relationships and classifications that were not explicitly stated in Semantic Web Rule Language rules but followed logically from the ontology’s axioms and class definitions. For instance, if a patient was inferred to have “Severe_Sensorineural_HL” and the ontology axiomatically defines that severe sensorineural hearing loss is a subclass of hearing loss requiring amplification, the reasoner automatically infers candidacy for hearing aids or cochlear implants without requiring explicit rules for every possible severity-treatment combination.

### 4.6. Integration with SNOMED CT and Interoperability

The alignment with SNOMED CT terminology provides substantial advantages for clinical implementation and data integration. SNOMED CT is internationally recognized and adopted in numerous countries’ national health information infrastructure initiatives [30], making our ontology compatible with global healthcare interoperability standards. The direct incorporation of SNOMED CT hierarchy into our ontology enables automatic translation of our classification results into standardized codes that can be recorded in electronic health records, shared across institutions, used for epidemiological reporting, and potentially integrated into billing and reimbursement systems where applicable.

During the mapping process, we successfully aligned core hearing loss concepts with SNOMED CT codes, ensuring that diagnoses generated by our system correspond to standardized terminology recognized across healthcare systems. For concepts lacking direct SNOMED CT equivalents, we retained our domain-specific classifications while documenting the mapping gap, enabling future integration as SNOMED CT continues to evolve and expand its audiology coverage. This pragmatic approach balances the need for standardization with the requirement to capture all clinically relevant nuances in our domain-specific application.

### 4.7. Limitations and Considerations

Several methodological limitations warrant acknowledgment and suggest directions for future research. First, the dataset derives exclusively from adult participants in the 2015–2016 National Health and Nutrition Examination Survey cycle, potentially limiting generalizability to pediatric populations where hearing loss etiologies, diagnostic criteria, and treatment approaches differ substantially [55]. Pediatric hearing loss frequently involves developmental considerations, genetic factors, and different severity distributions compared to adult populations. Multi-institutional validation studies including both adult and pediatric cohorts from diverse geographic regions and healthcare systems are necessary to assess external validity and identify potential performance degradation in populations with different demographic characteristics or hearing loss prevalence patterns.

Second, while the National Health and Nutrition Examination Survey dataset provides comprehensive audiometric and medical data, it lacks certain potentially valuable information, such as genetic testing results, detailed occupational noise exposure histories with quantitative measurements, imaging findings from temporal bone computed tomography or magnetic resonance imaging, and longitudinal outcome data that could further refine classification and treatment prediction. Future iterations could incorporate these additional data modalities to enhance predictive accuracy and clinical utility.

Third, the computational complexity of ontological reasoning with Description Logic inference engines may limit real-time applications in high-throughput clinical settings without further optimization [46]. While reasoning over individual patient cases is computationally feasible (typically requiring seconds to minutes on standard hardware), batch processing of large patient populations or integration into systems requiring immediate real-time responses may necessitate optimization strategies such as pre-computed inference results, incremental reasoning approaches, or hybrid architectures that combine offline ontology reasoning with online machine learning prediction.

Fourth, this study focuses on classification of hearing loss types and treatment recommendations but does not address all aspects of comprehensive audiology care, such as hearing aid fitting parameters, specific counseling approaches, or monitoring protocols for progressive hearing loss. Extending the ontology and predictive models to encompass these additional clinical tasks would enhance practical utility for complete clinical workflows.

Fifth, the ontology was developed primarily by the research team with input from the domain literature rather than through extensive collaborative ontology engineering with large panels of practicing audiologists from diverse clinical settings. While the ontology incorporates established clinical knowledge and SNOMED CT alignment ensures terminological validity, future work would benefit from broader expert validation and potential refinement through Delphi methodology or similar consensus approaches to ensure clinical acceptability across diverse practice settings and specialties.

Also, while post-hoc explanations were provided for the observed increase in log loss relative to accuracy, formal calibration analysis of the Random Forest model was not conducted in this study. Future work will include calibration assessments, such as reliability diagrams and Brier scores, to ensure that predicted probabilities are accurate and interpretable for clinical decision-making.

Finally, although our results demonstrate strong classification performance on retrospectively collected data, we have not yet conducted prospective clinical validation studies comparing automated classifications against expert audiologist diagnoses in real-world clinical workflows. Moreover, the reported performance improvements (approximately 2%) were not subjected to formal statistical significance testing or confidence interval estimation. Consequently, these gains should be interpreted as indicative rather than conclusive. Future work should incorporate rigorous statistical analyses, such as bootstrapped confidence intervals or paired hypothesis tests, as well as prospective clinical validation, to robustly quantify the significance and reproducibility of the observed improvements.

### 4.8. Future Research Directions

Future investigations should address identified limitations and explore promising extensions of the hybrid ontology-machine learning framework. First, expanding the patient dataset to include pediatric populations, longitudinal follow-up data tracking hearing loss progression and treatment outcomes, and multi-institutional cohorts from diverse healthcare settings would enhance generalizability assessment and enable the development of age-specific and context-specific models. Second, incorporating additional data modalities, such as genetic information (mutations in *GJB2* or other deafness-related genes); imaging findings from temporal bone computed tomography or magnetic resonance imaging showing anatomical abnormalities; electrophysiological tests, including auditory brainstem response and otoacoustic emissions; and patient-reported outcome measures assessing quality of life and functional hearing, could refine diagnostic accuracy and enable more personalized treatment recommendations. Third, exploring advanced machine learning techniques, including deep learning architectures (recurrent neural networks for temporal audiometric data, convolutional neural networks for spectrogram analysis, transformers for multimodal data fusion) or gradient boosting methods (XGBoost 1.7.6, LightGBM 3.3.5), might further improve predictive performance while maintaining interpretability through attention mechanisms or integrated gradient analysis.

Fourth, extending the ontology to capture treatment outcomes, adverse effects, patient adherence patterns, and longitudinal progression trajectories would enable prognostic modeling and treatment effectiveness prediction, moving beyond diagnostic classification toward comprehensive clinical decision support throughout the patient care continuum. Fifth, developing user-friendly clinical interfaces that present ontology-derived explanations and machine learning predictions in intuitive formats suitable for practicing audiologists would facilitate translation from research prototypes to deployable clinical tools. Usability testing and iterative refinement based on clinician feedback would be essential for successful implementation. Finally, investigating federated learning approaches could enable collaborative model development across multiple institutions while preserving patient privacy and complying with data protection regulations, addressing the challenge of developing robust models on large, diverse datasets without centralizing sensitive health information.

## 5. Conclusions

This study presents a novel hybrid framework integrating SNOMED CT-aligned clinical ontology with Random Forest machine learning to advance hearing loss diagnosis and personalized treatment recommendations. Developed and validated on 3723 adult patients from the National Health and Nutrition Examination Survey dataset, the model achieved a classification accuracy of 92.48% for hearing loss types (normal, conductive, sensorineural, mixed) with corresponding treatment recommendations, substantially surpassing the K-Means clustering baseline by 2.28 percentage points. The ontology, utilizing Semantic Web Rule Language rules executed by the Pellet 2.2.0 reasoner, facilitated precise semantic labeling and feature augmentation, significantly enhancing the model’s ability to capture complex clinical patterns, as evidenced by improved F1-scores across all classes, with particularly notable gains for mixed hearing loss (0.86 to 0.92) and normal hearing (0.85 to 0.89). This hybrid approach outperforms existing machine learning models in audiology, which typically achieve accuracies between 85% and 91% without semantic integration, and extends purely ontology-based systems by incorporating actionable predictive analytics for clinical decision support.

The methodology’s alignment with SNOMED CT ensures semantic interoperability with global medical standards and electronic health record systems, enabling seamless integration into existing clinical workflows and facilitating automated data exchange across healthcare institutions. Feature importance analysis identified audiometric thresholds at high frequencies, ontology-derived severity labels, and medical history variables as primary predictors, enhancing clinical interpretability and supporting evidence-based decision-making. These attributes position the hybrid framework as a robust and transparent decision-support tool for audiologists, directly addressing the critical need for personalized interventions in hearing loss management. The cross-validation results (mean accuracy 92.80%, standard deviation 0.9%) demonstrate strong generalization capability and model stability, providing confidence in performance reliability across diverse patient populations.

Several limitations warrant acknowledgment, including the dataset’s focus on adult populations, which constrains applicability to pediatric hearing loss, the computational demands of ontological reasoning, which may require optimization for high-throughput clinical deployment, the absence of certain data modalities such as genetic and imaging information, and the lack of prospective clinical validation comparing automated recommendations against expert audiologist assessments. Despite these limitations, the demonstrated performance and clinical interpretability suggest immediate applicability as a decision-support tool in audiology practice, particularly for complex or ambiguous cases where structured reasoning may assist clinician judgment.

Future investigations should expand the patient cohort to include pediatric populations and longitudinal follow-up data, incorporate additional data modalities such as genetic information and imaging findings to refine diagnostic precision, explore advanced machine learning architectures including deep learning while maintaining interpretability, extend the ontology to capture treatment outcomes and adverse effects for prognostic modeling, develop intuitive clinical interfaces for practicing audiologists, and investigate federated learning approaches to enable collaborative model development across institutions while preserving patient privacy. 

In conclusion, this study establishes a rigorous foundation for knowledge-driven artificial intelligence in audiology, demonstrating the efficacy of hybrid semantic-predictive systems in precision medicine. The framework provides a scalable and adaptable model for advancing personalized healthcare through principled integration of domain knowledge with data-driven analytics, with potential applicability beyond audiology to other medical specialties facing similar challenges in diagnosis and treatment personalization. However, based on the above-mentioned shortcomings, future high-quality research is warranted.

## Figures and Tables

**Table 1:** Ontology overview.

Aspects	Subaspects	Metrics
Classes	Superclasses Subclasses	9 63
Properties	Object properties Data properties	27 39
Axioms	-	145,683
Individuals	-	3723
Rules	SWRL rules	18

**Table 2:** Random Forest performance metrics by hearing loss (HL) class.

Hearing Loss Class	Precision	Recall	F1-Score
Sensorineural HL	0.91	0.89	0.91
Conductive HL	0.85	0.90	0.87
Mixed HL	0.86	0.86	0.86
Normal Hearing	0.87	0.86	0.85

**Table 3:** Random Forest performance metrics after ontology-based semantic enrichment.

Hearing Loss (HL) Class	Precision	Recall	F1-Score
Normal Hearing	0.94	0.91	0.93
Sensorineural HL	0.92	0.93	0.92
Conductive HL	0.89	0.90	0.89
Mixed HL	0.92	0.91	0.92

**Table 4:** Comparative Performance of Random Forest (RF) Models with K-Means Clustering and Ontology-Based Enrichment.

Metric	RF + K-Means	RF + Ontology
Test set accuracy (%)	90.2	92.48
Cross-validation mean accuracy (%)	91.22	92.80
Cross-validation Standard Deviation (%)	1.2	0.9
F1-score–Sensorineural Hearing Loss (HL)	0.91	0.92
F1-score–Conductive HL	0.87	0.89
F1-score–Mixed HL	0.86	0.92
F1-score–Normal Hearing	0.85	0.89
Log-loss	0.2766	0.3842

## Data Availability

The National Health and Nutrition Examination Survey 2015–2016 data used in this study are publicly available from the Centers for Disease Control and Prevention National Center for Health Statistics website (https://wwwn.cdc.gov/nchs/nhanes/) (accessed on 29 October 2025). The developed ontology and analysis code are available upon reasonable request from the corresponding authors.
